# Tumor treating fields combined with concurrent chemoradiotherapy for glioblastoma: A multi-institutional analysis

**DOI:** 10.1016/j.isci.2026.116419

**Published:** 2026-06-23

**Authors:** Menglan Zhai, Guangyuan Hu, Guoping Shen, Yahua Zhong, Jing Huang, Qianxia Li, Liping Liang, Yong Huang, Jianping Bi, Ying Li, Yanping Li, Guoliang Pi, Hongwei Shi, Hanping He, Vivek Verma, Yang Wang, Guang Han

**Affiliations:** 1 Department of Radiation Oncology, Hubei Cancer Hospital, Tongji Medical College, Huazhong University of Science and Technology, Wuhan, China; 2 Department of Oncology, Tongji Hospital, Tongji Medical College, Huazhong University of Science and Technology, Wuhan, China; 3 Department of Radiation Oncology, the first Affiliated Hospital of Sun Yat-sen University, Guangzhou, China; 4 Department of Radiation and Medical Oncology, Zhongnan Hospital, Wuhan University, Wuhan, China; 5 Cancer Center, Union Hospital, Tongji Medical College, Huazhong University of Science and Technology, Wuhan, China; 6 Department of Radiation Center, Huashan Hospital, Fudan University, Shanghai, China; 7 Department of Radiation Oncology, the University of Texas MD Anderson Cancer Center, Houston, TX, USA

**Keywords:** oncology, therapeutics

## Abstract

The clinical benefit of extending tumor treating fields (TTFields) beyond concurrent chemoradiotherapy (CRT) for newly diagnosed glioblastoma (ndGBM) is unclear. This multi-institutional retrospective study compared patients who continued TTFields into adjuvant temozolomide (CRT-TT, *n* = 68) versus those who discontinued after CRT (CRT-T, *n* = 32). With a median follow-up of 36.9 months, median progression-free survival (mPFS) did not significantly differ between the groups (12.8 vs. 12.5 months; hazard ratio [HR] 0.95, 95% confidence interval [CI] 0.57–1.59; *p* = 0.853) nor did median overall survival (mOS) (20.6 vs. 16.6 months; HR 0.73, 95% CI 0.43–1.27; *p* = 0.267). Additionally, continuing TTFields into the adjuvant phase did not increase scalp toxicity. Although a numerically longer OS was seen in the CRT-TT group, the lack of statistical significance may reflect limited power and non-randomized allocation. These findings underscore the need for further investigation.

## Highlights

• 100 ndGBM patients treated with TTFields were recruited from six cancer centers
• Among them, 68 patients continued TTFields into adjuvant TMZ and 32 discontinued after CRT
• Extended TTFields showed no significant survival difference or increased scalp toxicity
• These preliminary real-world finings underscore the need for further investigation

## Graphical abstract

**Figure:**
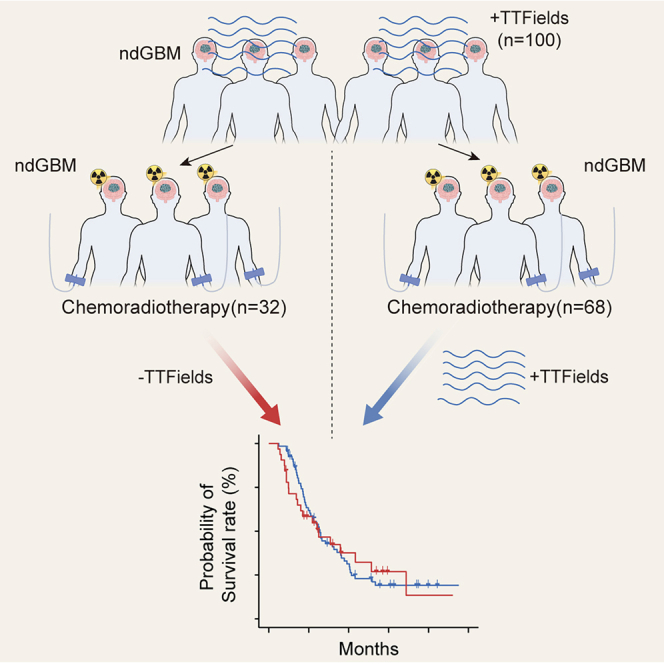

## Introduction

Glioblastoma (GBM), the most common malignant central nervous system tumor in adults, is characterized by high aggressiveness and a propensity for recurrence.^1^^,^^2^ Despite treatment with maximal safe surgical resection combined with radiotherapy (RT) and chemotherapy, the 5-year survival rate remains merely 5.8%.^3^^,^^4^ Tumor treating fields (TTFields), a novel non-invasive antimitotic treatment modality, utilizes low-intensity (1–3 V/cm), intermediate-frequency (100–300 kHz) alternating electric fields to disrupt neoplastic cell division and inhibit proliferation.^5^ In the global phase 3 EF-14 study, the addition of TTFields to adjuvant temozolomide (TMZ) significantly enhanced progression-free survival (PFS) and overall survival (OS) in patients with newly diagnosed GBM (ndGBM) compared to adjuvant TMZ alone, with an increase in median PFS (mPFS) from 4.0 months to 6.7 months and median OS (mOS) from 16.0 months to 20.9 months.^6^^,^^7^ Based on this study, the US Food and Drug Administration (FDA) approved TTFields in combination with TMZ for the treatment of glioblastoma. Despite this breakthrough, there remain many unmet needs for this patient population.

Mechanistically, TTFields also enhance radiosensitivity by delaying DNA damage repair and increasing DNA replication pressure.^5^^,^^8^ This provides a theoretical basis for the application of TTFields in combination with RT. To test this, Bokstein et al. prospectively evaluated the safety and feasibility of TTFields/RT/TMZ followed by adjuvant TMZ/TTFields in ndGBM. Results showed a mPFS of 8.9 months (95% confidence interval [CI]: 2.1–12.9), superior to the 6.7 months (95% CI: 6.1–8.1) in the EF-14 study, with mOS not yet reached. Although most patients (8/10) experienced grade 1–2 scalp toxicity, no other TTFields-related adverse events (AEs) were observed.^9^ With the same treatment paradigm, the SPARE study also observed mPFS of 9.3 months and mOS of 15.8 months, and no grade 3 or higher TTFields-related AEs were observed.^10^ These results demonstrated the feasibility and acceptable safety profile of combining TTFields with RT and TMZ in patients with ndGBM.

However, the aforementioned trials were small (*n* = 10 and *n* = 30, respectively) and as such this therapeutic paradigm requires data from larger cohorts. The clinical rationale for delivering concurrent TTFields with chemoradiotherapy (CRT) in routine practice is supported by both mechanistic and emerging clinical evidence. Preclinical studies have demonstrated that TTFields enhance tumor radiosensitivity by delaying DNA damage repair and increasing DNA replication pressure,^5^^,^^8^ providing a strong biological rationale for combining TTFields with RT. Furthermore, the safety and feasibility established by these small prospective studies led participating institutions to adopt this combination as an evidence-based, off-label clinical care option for eligible patients with isocitrate dehydrogenase (IDH)-wildtype tumors, good performance status, and no contraindications to TTFields device use during the study enrollment period (2020–2024). All treatment decisions were made by the multidisciplinary teams at each institution after informed discussion with patients, reflecting real-world clinical practice. Key practical questions regarding the optimal duration of therapy remain unresolved. Standard adjuvant TMZ is typically administered for 6 to 12 cycles, while TTFields in the EF-14 trial were continued until disease progression or for up to 24 months. However, the potential value of extending TTFields beyond the concurrent chemoradiation phase remains unknown, a question that the EF-14 trial was not designed to address.^6^^,^^7^ This uncertainty may influence clinical decision-making, patient adherence, and health care resource utilization. An international phase 3 randomized trial (EF-32, NCT04471844) has completed enrollment of 981 patients and is designed to evaluate the efficacy of concurrent plus adjuvant TTFields with CRT.^11^ Against this background, the present multi-institutional real-world study was performed to explore this relevant clinical question.

## Results

### Patient characteristics

A total of 100 patients treated with TTFields were recruited from six cancer centers. Median follow-up was 36.9 months (95% CI: 33.5–40.3), at which time 34 patients remained alive; seven patients were still receiving TTFields therapy. The detailed baseline demographics of the enrolled patients are presented in Table 1. The median age was 51 years, 59% were men, and the median Karnofsky performance score (KPS) was 80. Only 4% of cases were in subtentorial region. Seventy-two percent of patients underwent gross total tumor resection (as assessed by intra-operative evaluation and confirmed on post-operative imaging), and 8% underwent diagnostic biopsy only. All patients had (IDH)-wildtype tumors, with unmethylated O(6)-methylguanine-DNA-methyltransferase (MGMT) promoter status being the most predominant molecular profile.

**Table 1:** Patient characteristics

Characteristics	No. (%) of patients		*P* value
	CRT-TT (*n* = 68)	CRT-T (*n* = 32)	
Sex			0.209
Male	43 (63)	16 (50)	
Female	25 (37)	16 (50)	
Age, years			0.891
≤51	35 (52)	16 (50)	
>51	33 (49)	16 (50)	
Baseline KPS score			0.607
<80	24 (35)	13 (41)	
≥80	44 (65)	19 (60)	
Tumor location			1.000
Supratentorial	65 (96)	31 (97)	
Subtentorial	3 (4)	1 (3)	
Resection			0.985
Gross total	49 (72)	23 (72)	
Partial/Biopsy	19 (28)	9 (28)	
MGMT methylation			0.073
Unmethylated	43 (63)	17 (53)	
Methylated	21 (31)	8 (25)	
Unknown	4 (6)	7 (22)	
Array status of RT period			0.124
Take off the array	15 (22)	3 (9)	
Keep the array	53 (78)	29 (91)	
Mean patient compliance with TTFields median (range), hours/day	20.4 (11.0–23.0)	20.0 (8.9–22.1)	0.174

MGMT, O(6)-methylguanine-DNA-methyltransferase; KPS, Karnofsky performance score; RT, radiotherapy; TTFields, tumor treating fields.

Eighteen patients had TTFields arrays removed daily with radiation delivery, and the remaining patients retained the TTFields arrays during RT. The median daily compliance during the concurrent phase was 20.4 h in the CRT-TT group and 20.0 h in the CRT-T group, with no significant difference between groups (*p* = 0.62). The median duration of treatment with TTFields in the CRT-TT group was 10.1 months.

### Reasons for TTFields discontinuation after concurrent CRT

Among the 32 patients who discontinued TTFields after completing RT (CRT-T group), the primary reasons for discontinuation were as follows. Patient-reported adherence burden was the most common reason, accounting for 44% of discontinuations (*n* = 14). These patients reported difficulty with long-term daily device use (≥18 h/day) due to impacts on daily living, social activities, or quality of life. Financial constraints accounted for 28% of discontinuations (*n* = 9), as self-paying patients could not sustain the ongoing costs of device rental and array replacement for the adjuvant phase. Mild scalp toxicity led to discontinuation in 16% of patients (*n* = 5), who experienced persistent grade 1–2 scalp toxicity despite local management and elected to discontinue TTFields to resolve skin symptoms. No patients discontinued due to high-grade (grade ≥3) toxicity. Treating physician recommendation accounted for 9% of discontinuations (*n* = 3), based on individualized factors such as performance status decline, comorbidities, or early signs of non-response. Other reasons accounted for 3% of discontinuations (*n* = 1), specifically relocation to a region without access to TTFields clinical monitoring.

Notably, no patients in the CRT-T group discontinued TTFields due to disease progression at the time of CRT completion. All patients in the CRT-T group were progression-free at the end of radiation, a criterion that was incorporated into the patient inclusion criteria to minimize immortal time bias.

### Efficacy outcomes

**Figure 1:**
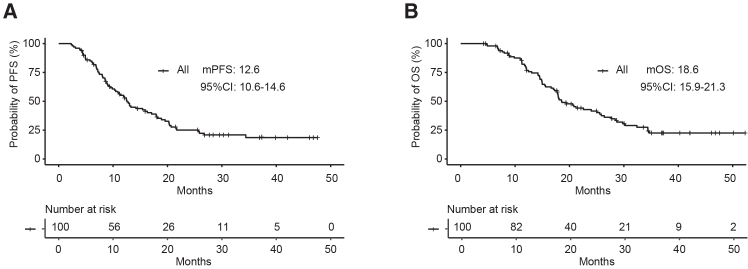
Kaplan-Meier survival curves for patients with ndGBM Kaplan-Meier survival curves for (A) progression-free survival (PFS) and (B) overall survival (OS) in 100 patients with ndGBM.

The mPFS for the entire population was 12.6 months (95% CI: 10.6–14.6) and the mOS was 18.6 months (95% CI: 15.9–21.3). The 1-year PFS and OS rates were 54% and 79%, respectively (Figure 1). A sensitivity landmark analysis performed at 3 months post-baseline to address potential guarantee-time bias, excluding 3 patients with early progression (*n* = 97), yielded consistent results with no statistically significant difference in PFS (*p* = 0.927) or OS (*p* = 0.350) between the treatment strategies (Figure S1).

**Figure 2:**
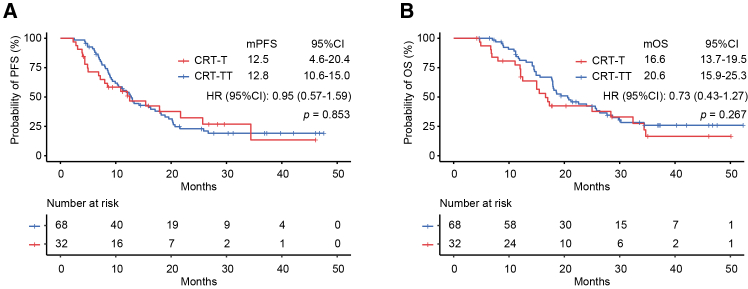
Kaplan-Meier survival comparison between two treatment cohorts Kaplan‑Meier comparison of (A) progression-free survival (PFS) and (B) overall survival (OS) between the CRT‑TT and CRT‑T groups.

With regard to adjuvant management after RT/TMZ completion, 68 received adjuvant TMZ/TTFields (CRT-TT group), and 32 received adjuvant TMZ (CRT-T group). No significant difference in PFS was observed between the CRT-TT and CRT-T groups (mPFS: 12.8 vs. 12.5 months; hazard ratio [HR]: 0.95; 95% CI: 0.57–1.59; *p* = 0.853) (Figure 2A). Similarly, no significant intergroup difference was observed in mOS, with 20.6 months (95% CI: 15.9–25.3) for the CRT-TT group compared to 16.6 months (95% CI: 13.7–19.5) for the CRT-T group (HR: 0.73; 95% CI: 0.43–1.27; *p* = 0.267) (Figure 2B). The 1-year PFS and OS rates were 54% and 81%, respectively, in the CRT-TT group, compared with 55% and 74% in the CRT-T group (Figure 2).

**Figure 3:**
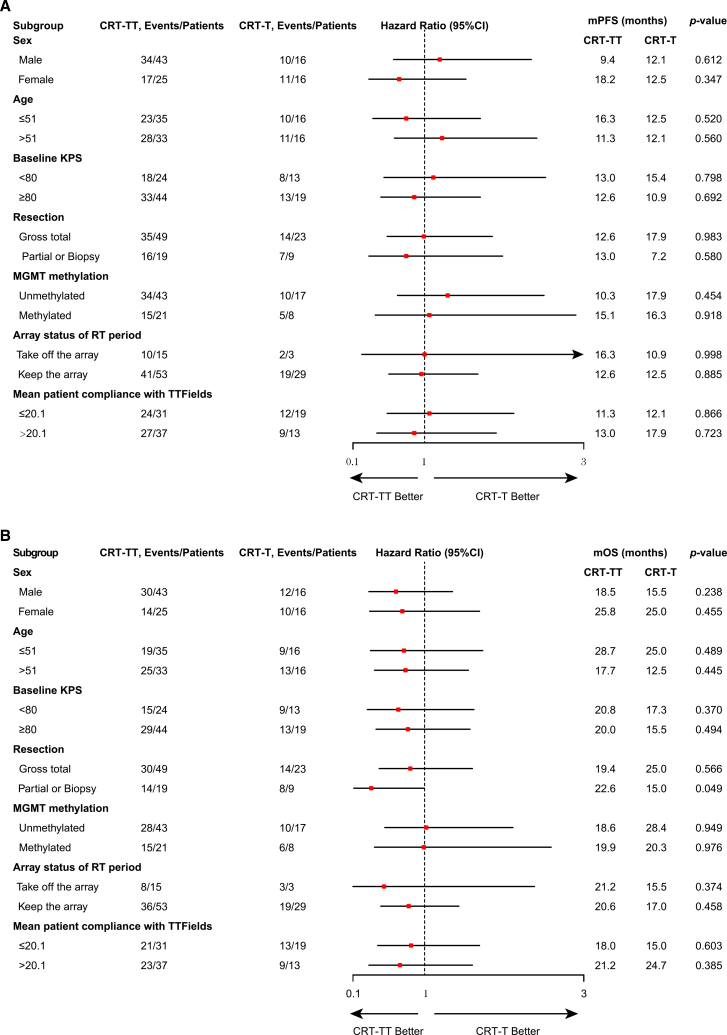
Forest plot of subgroup survival analyses Forest plot of subgroup analysis for (A) progression-free survival (PFS) and (B) overall survival (OS).

For subgroup analysis, the following variables were evaluated: sex, age, baseline KPS score, extent of resection, MGMT promoter methylation status, electrode array removal during RT, and mean patient compliance with TTFields therapy. No significant differences in mPFS or mOS were observed between the CRT-TT and CRT-T groups across most subgroups, except for the extent of resection category (partial resection or biopsy) (Figure 3). Notably, among patients who underwent partial resection or biopsy, the CRT-TT group demonstrated longer mOS compared to the CRT-T group (22.6 months, 95% CI: 14.6–29.8 vs. 15.0 months, 95% CI: 4.7–28.4; *p* = 0.049). This significant interaction warrants further investigation but should be interpreted with caution given the lack of a significant difference in the primary analysis, the small sample size within this subgroup, and the potential influence of subsequent salvage therapies or tumor biology heterogeneity on post-progression survival.

Prognostic factors for PFS and OS in the entire cohort were evaluated using Cox regression (Tables 2 and 3). In univariate analysis, no variable was significantly associated with PFS, although age (>51 years) and partial resection/biopsy showed a trend toward worse PFS. For OS, older age (>51 years) was significantly associated with inferior survival in univariate analysis (HR = 2.21; *p* < 0.001). On multivariable analysis, age remained an independent prognostic factor for OS (HR = 2.35; *p* < 0.001). Multivariable Cox regression analysis, adjusting for age and extent of resection, confirmed no significant difference in PFS between the CRT-TT and CRT-T groups (aHR = 0.95, 95% CI 0.57–1.59; *p* = 0.853). Similarly, after adjustment for sex, age, and extent of resection, OS did not differ significantly between groups (aHR = 0.73, 95% CI 0.43–1.27; *p* = 0.267).

**Table 2:** Univariate and multivariable Cox analysis of PFS in the overall population

	Univariate analysis		Multivariate analysis	
	HR (95%CI)	*p* value	HR (95%CI)	*p* value
**Sex**				

Male	1	–	–	–
Female	0.70 (0.44–1.13)	0.142	–	–

**Age**				

≤51	1	–	–	–
>51	1.55 (0.97–2.48)	0.065	1.48 (0.93–2.38)	0.100

**Baseline KPS score**				

<80	1	–	–	–
≥80	1.10 (0.68–1.79)	0.689	–	–

**Extent of initial resection**				

Gross total resection	1	–	–	–
Partial resection or Biopsy	1.56 (0.95–2.57)	0.081	1.47 (0.89–2.44)	0.131

**MGMT promoter methylation**				

Unmethylated	1	–	–	–
Methylated	0.78 (0.46–1.32)	0.351	–	–

**Array status of RT period**				

Take off the array	1	–	–	–
Keep the array	0.90 (0.49–1.68)	0.747	–	–
Mean patient compliance with TTFields	1.06 (0.54–2.10)	0.858	–	–

**Treatment group**				

CRT-T	–	–	1	–
CRT-TT	–	–	0.95 (0.57–1.59)	0.853

PFS, progression-free survival; MGMT, O(6)-methylguanine-DNA-methyltransferase; KPS, Karnofsky performance score; RT, radiotherapy; TTFields, tumor treating fields.

**Table 3:** Univariate and multivariable Cox analysis of OS in the overall population

	Univariate analysis		Multivariate analysis	
	HR (95%CI)	*P* value	HR (95%CI)	*P* value
**Sex**				

Male	1	–	–	–
Female	0.63 (0.38–1.05)	0.076	0.61 (0.37–1.02)	0.057

**Age**				

≤51	1	–	–	–
>51	2.21 (1.34–4.55)	<0.001	2.35 (1.43–3.87)	<0.001

**Baseline KPS score**				

<80	1	–	–	–
≥80	1.05 (0.64–1.73)	0.853	–	–

**Extent of initial resection**				

Gross total resection	1	–	–	–
Partial resection or Biopsy	1.59 (0.95–2.66)	0.079	1.48 (0.89–2.48)	0.135

**MGMT promoter methylation**				

Unmethylated	1	–	–	–
Methylated	0.97 (0.57–1.66)	0.916	–	–

**Array status of RT period**				

Take off the array	1	–	–	–
Keep the array	0.96 (0.50–1.84)	0.899	–	–
Mean patient compliance with TTFields	0.65 (0.72–1.05)	0.264	–	–

**Treatment group**				

CRT-T	–	–	1	–
CRT-TT	–	–	0.73 (0.43–1.27)	0.267

OS, overall survival; MGMT, O(6)-methylguanine-DNA-methyltransferase; KPS, Karnofsky performance score; RT, radiotherapy; TTFields, tumor treating fields.

### Toxicity

Of the 100 patients, 76 (76%) developed TTFields-related skin AEs (Table 4). Grade 1–2 AEs predominated, occurring in 71% of the CRT-TT group and 78% of the CRT-T group (*p* = 0.428), while grade 3 scalp toxicity was observed in only 3% of patients. Notably, all three grade 3 events occurred during the adjuvant phase, with no patient experiencing grade 3 scalp toxicity during the period of concurrent RT and TTFields administration. Typical presentations included contact dermatitis, pruritus, erosions, blisters, seborrheic keratosis, folliculitis, electric sensation, and skin-burning sensations, all of which were adequately controlled with topical glucocorticoids or antibiotics.

**Table 4:** TTfields-related skin AEs in groups CRT-TT and CRT-T

Skin AEs	No. (%) of patients		*P* value
	CRT-TT (*n* = 68)	CRT-T (*n* = 32)	
Grade 0	19 (28)	5 (16)	0.179
Grade 1-2	48 (71)	25 (78)	0.428
Grade 3	1 (2)	2 (6)	0.239

During the concurrent phase, TTFields therapy was temporarily interrupted due to scalp toxicity in three patients per group. All interruptions lasted ≤7 days, and treatment was resumed after local management. No patient permanently discontinued TTFields during RT due to toxicity, and no balance disorders, falls, fractures, or central nervous system toxicities related to TTFields use were observed. TTFields compliance remained high throughout the concurrent phase, with median daily usage of 20.4 h in the CRT-TT group and 20.0 h in the CRT-T group (*p* = 0.62), well above the recommended 18 h per day for optimal efficacy. These findings demonstrate that concurrent administration of TTFields with RT did not compromise treatment adherence or introduce unexpected safety concerns.

## Discussion

This multicenter analysis evaluates the efficacy and safety of patients with ndGBM receiving TTFields and concurrent CRT and adjuvant TMZ, with or without TTFields maintenance therapy. The findings of this study confirm that TTFields combined with CRT is both effective and tolerable. A comparative analysis revealed that there was no statistically significant survival disparity between the CRT-TT and CRT-T groups. Our multicenter analysis (*n* = 100) demonstrates that synchronous TTFields application with concurrent CRT maintains a comparable grade 3 TTFields-related scalp toxicity profiles to the EF-14 trial’s TMZ/TTFields regimen (3% vs. 2%).^6^ In terms of efficacy, we observed a median PFS of 12.6 months, which compares favorably to historical benchmarks for standard concurrent CRT followed by adjuvant TMZ alone (6.9 months)^12^ and is markedly superior to the adjuvant-only control arm of the EF-14 trial (6.7 months).^6^ The median OS of 18.6 months in our cohort also appears favorable compared to smaller previous studies of concurrent TTFields, such as Bokstein et al.^9^ (mOS not reached) and the SPARE trial (mOS: 15.8 months).^10^

Importantly, this analysis provides evidence suggesting that patients who received TTFields only during concurrent CRT had survival outcomes that were not statistically significantly different from those who continued treatment into the adjuvant phase. A retrospective study in China investigated a similar treatment paradigm, comparing the CRT-TT approach to concurrent CRT followed by adjuvant TMZ/TTFields, and the primary analysis failed to demonstrate significant improvements in PFS or OS between treatment arms.^13^ However, consistent with the results of this study, a survival benefit emerged through post hoc analysis in a specific subgroup of patients with subtotal resection only or biopsy only. All subgroup analyses presented in this study should be considered exploratory, as they were not pre-specified and are subject to the limitations of multiple comparisons and reduced statistical power within smaller strata. The observed interaction in the partial resection/biopsy subgroup, while suggestive, requires validation in larger, prospectively designed studies. These findings suggest that prolonged TTFields application may not be the optimal treatment strategy for all patients with ndGBM, and that concurrent TTFields could be a critical radiosensitization window for RT. In addition, given the substantial cost implications of prolonged TTFields use,^14^ restricting TTFields to the CRT phase may be a feasible option.

Building on these efficacy findings, the safety profile observed in our cohort aligns with and extends the findings from earlier smaller studies that first established the feasibility of concurrent TTFields with RT. Bokstein et al. reported grade 1–2 scalp toxicity in 8 of 10 patients with no grade ≥3 events during concurrent TTFields and RT,^9^ while the SPARE trial observed no grade ≥3 TTFields-related adverse events in 30 patients receiving this combination.^10^ Our multi-institutional cohort of 100 patients corroborates these findings, demonstrating that grade 3 scalp toxicity occurred in only 3% of patients, a rate comparable to the 2% reported in the EF-14 trial for adjuvant-only TTFields,^6^ and that these events were not confined to the concurrent phase. The predominance of manageable grade 1–2 scalp toxicity, the absence of other serious TTFields-related AEs, and the high treatment compliance during RT collectively reinforce the conclusion that concurrent TTFields and RT is a well-tolerated regimen suitable for broader clinical application.

A key question arises when considering our results alongside the EF-14 trial: if TTFields combined with adjuvant TMZ improved survival in EF-14, why does extending TTFields beyond concurrent CRT in our cohort not show a significant additional benefit? First and most importantly, our study was underpowered to detect a moderate effect size. With only 68 patients in the CRT-TT group and 32 in the CRT-T group, the statistical power to detect a 4-month difference in mOS (HR 0.73) was far below conventional thresholds, meaning that a true benefit could easily have been missed. Thus, the absence of a statistically significant difference does not imply equivalence and likely reflects inadequate sample size rather than a true lack of benefit. Second, the radiosensitizing effect of TTFields during CRT may constitute a uniquely potent therapeutic window, maximizing initial cytoreduction and potentially diminishing the relative incremental value of prolonged adjuvant therapy. This hypothesis aligns with preclinical evidence demonstrating TTFields enhance radiosensitivity by impairing DNA damage repair.^5^^,^^8^ Furthermore, the observed numerical difference in mOS (20.6 vs. 16.6 months) should be interpreted with caution. OS in glioblastoma is heavily influenced by post-progression therapies (e.g., bevacizumab, re-irradiation, second-line chemotherapy, or TTFields re-challenge) and socioeconomic factors that affect access to salvage treatments. In our cohort, patients who continued TTFields into the adjuvant phase may have had better financial support, which could confound the OS comparison. In contrast, the nearly identical mPFS between the two groups (12.8 vs. 12.5 months) suggests that the radiosensitizing effect of concurrent TTFields may be the dominant determinant of progression control, with limited incremental benefit from extended adjuvant TTFields on PFS.

Beyond the timing of TTFields, the observed survival pattern likely reflects the characteristically aggressive biology of recurrent GBM and the limited efficacy of salvage therapies. Clonal evolution after CRT might select for TTFields-resistant populations, and the burden of long-term device use may also influence outcomes, confounders that are difficult to capture retrospectively. Lastly, our reliance on conventional biomarkers (IDH and MGMT status) meant that comprehensive molecular profiling was unavailable, thereby limiting our ability to discern biological subsets that may respond differently to the duration of TTFields therapy. Thus, while our study cannot answer these mechanistic questions, it highlights the necessity of incorporating translational biomarkers in future prospective trials.

In conclusion, this real-world study evaluates the efficacy and safety of patients with ndGBM receiving TTFields, concurrent CRT and adjuvant TMZ, with or without TTFields maintenance therapy. The findings revealed comparable rates of scalp toxicity between the CRT-TT and CRT-T groups, with no statistically significant differences observed in mPFS or mOS. The findings of this study highlight the uncertainty surrounding the incremental benefit of extending TTFields beyond concurrent CRT. Further research is warranted to clarify the optimal treatment duration.

### Limitations of the study

The most significant limitation of our study is the non-randomized allocation to treatment groups, which introduces selection bias and limits causal inference. Additionally, the sample size provides insufficient power to detect moderate effect sizes; for example, a randomized trial designed to confirm the observed difference in mOS would require a substantially larger cohort. Therefore, our null findings do not rule out a genuine benefit of extending TTFields beyond CRT. In light of the EF-32 design, we do not advocate for an equivalency trial comparing concurrent + adjuvant versus concurrent-only, as such a trial would be logistically challenging and unlikely to alter clinical practice if concurrent + adjuvant proves superior to adjuvant only. Given the limitations of individual retrospective studies, we acknowledge that an individual patient data (IPD) meta-analysis of existing real-world cohorts (including the present study and other published series) could increase statistical power and allow subgroup analyses to better define the potential benefit of extended TTFields. Such an approach may provide practice-informing evidence while awaiting the results of the EF-32 trial. Instead, future research should focus on identifying predictive biomarkers (e.g., MGMT methylation status, residual tumor volume) that may help select patients most likely to benefit from extended TTFields, and on strategies to improve long-term adherence.

## Resource availability

### Lead contact

Further information and requests for resources should be directed to and will be fulfilled by the lead contact, Guang Han (hg7913@hotmail.com).

### Materials availability

This study did not generate any new unique reagents or materials.

### Data and code availability

• The clinical data reported in this paper will be shared by the lead contact upon request.
• This paper does not report original code.
• Any additional information required to reanalyze the data reported in this paper is available from the lead contact upon request.

## Acknowledgments

This work was supported by PARP Inhibitor Oncology Research Funding of China Anti-Cancer Association (CACA) (CETSDHRCORP252-4-004), 10.13039/501100003819Hubei Provincial Natural Science Foundation Joint Fund Key Project - Hengrui Medical Innovation and Development Joint Fund (JCZRLH202500326), Scientific Research Projects of Hubei Cancer Hospital (2025HBCHYN07), Talent Project of Hubei Cancer Hospital (2025HBCHQHRC019), and Bejing Xisike Clinical Oncology Research Foundation (Y-HR2022QN-0488).

## Author contributions

Conceptualization, Y.Z., G.S., G. Hu, Y.W., and G. Han; data curation, M.Z., Q.L., L.L., Y.H., J.H., and G.S.; formal analysis, M.Z., Q.L., Y.H., J.H., and V.V.; funding acquisition, J.B. and G. Han; investigation, J.B., Ying Li, Yanping Li, G.P., H.S., and V.V.; software, J.B.; methodology, Ying Li, Y.Z., G. Hu, and Y.W.; project administration, H.H.; supervision, H.H., Y.Z., G.S., G. Hu, Y.W., and G. Han; writing – original draft, M.Z., Q.L., L.L., and Y.H.; writing – review and editing, M.Z., L.L., Y.H., and J.H. All authors read and approved the final manuscript.

## Declaration of interests

The authors declare no competing interests.

## STAR★Methods

### Key resources table

**Table:** 

REAGENT or RESOURCE	SOURCE	IDENTIFIER
**Deposited data**		

Raw data	This paper	N/A

**Software and algorithms**		

Software for statistical analysis	R studio	v4.4.1
Software for statistical analysis	IBM SPSS	V27.0

### Experimental model and study participant details

#### Ethics approval

This study was performed in accordance with the Declaration of Helsinki. Ethical approval was obtained from the Ethics Committee of Hubei Cancer Hospital of Huazhong University of Science and Technology (approval number: LLHBCH2025YN-030). The other five participating institutions (Wuhan Union Hospital, Wuhan Tongji Hospital, Zhongnan Hospital of Wuhan University, Huashan Hospital, and the First Affiliated Hospital of Sun Yat-sen University) did not require separate ethical approval because, according to their local policies, retrospective studies using de-identified clinical data are exempt from formal review. Informed consent was waived owing to the retrospective nature of the study.

#### Study population

This retrospective study included patients with ndGBM who received TTFields and concurrent CRT between January 2020 and June 2024 at six hospitals (Hubei Cancer Hospital, Wuhan Union Hospital, Wuhan Tongji Hospital, Zhongnan Hospital of Wuhan University, Huashan Hospital, and the First Affiliated Hospital, Sun Yat-sen University). The inclusion criteria were as follows: (1) newly diagnosed and histologically confirmed GBM after primary surgical resection or biopsy; (2) completed the standard concurrent CRT phase (RT with concurrent TMZ); (3) initiated TTFields during concurrent CRT. Detailed demographic and baseline clinical information of the 100 enrolled patients can be found in Table 1.

### Method details

#### Treatment costs and insurance coverage

TTFields therapy costs were covered through various mechanisms reflecting real-world clinical practice in China. A subset of patients received coverage through provincial medical insurance schemes (e.g., Hubei, Guangdong) or commercial supplementary insurance, which partially or fully covered the costs of the device and transducer arrays. The majority of patients, however, funded treatment through out-of-pocket payments, a common scenario for novel anticancer therapies in real-world settings, particularly for off-label use.

#### Treatment

TTFields therapy was administered using the NovaTTFields-200A device (Novocure Ltd., Haifa, Israel). Low-intensity (2V/cm), intermediate frequency (200 kHz), and alternating electric fields were precisely delivered to the tumor regions through two orthogonally positioned transducer arrays. TTFields therapy was initiated within one week before or after the start of RT. All patients were recommended to receive TTFields for at least 18 hours daily to achieve optimal therapeutic outcomes. TTFields compliance data (hours/day) were obtained from device usage logs provided by the manufacturer. For each patient, mean daily compliance was calculated over the entire treatment period, defined as the interval from TTFields initiation to permanent discontinuation. Missing usage data occurred infrequently (<5% of scheduled downloads), typically due to technical download failures; in such instances, the last available recorded value was carried forward. No patient was excluded from analysis due to missing compliance data.

All treatments described in this study were delivered as standard real-world clinical care, not within the context of a prospective clinical trial. The decision to administer concurrent TTFields with CRT was made by the treating neuro-oncology and radiation oncology teams at each participating institution, based on individualized patient assessments and after informed discussion with patients and families regarding the potential benefits and risks of this off-label combination therapy, which was supported by emerging preclinical and clinical evidence available during the study period (2020 to 2024).

The decision to remove or retain transducer arrays during daily RT fractions was based on the specific protocol of each treating institution and the assessment of the radiation oncology team. Some centers adopted the practice of removing arrays to preclude any potential dosimetric interference and to optimize patient comfort during setup. In contrast, other centers retained the arrays on the patient’s scalp during daily RT fractions for convenience (avoiding reapplication), but the TTFields device was turned off while the radiation beam was on. TTFields therapy was resumed immediately after each RT fraction. This approach was adopted to maximize overall daily TTFields exposure time without delivering concurrent electrical fields during active irradiation, as no safety data exist for simultaneous application. Both approaches are considered clinically acceptable, and this procedural variation is characteristic of multi-institutional, real-world practice.

All patients received intensity-modulated radiotherapy (IMRT). IMRT was delivered as 1.8-2.0 Gy/fraction/day for 30 fractions delivered 5 consecutive days per week. Oral TMZ (75 mg/m^2^/day) was initiated on the same day as RT and continued throughout the entire RT course. During the adjuvant treatment phase, TMZ was orally administered at a daily dose of 150-200 mg/m^2^ for 5 consecutive days and repeated every 28 days, until 6-12 cycles were completed or disease progression, intolerable toxicity, patient withdrawal, or death occurred.

Patients were categorized into two cohorts based on the duration of TTFields therapy during the adjuvant phase. The abbreviations used are defined as follows: “CRT” denotes the initial treatment phase of concurrent chemoradiotherapy (RT with TMZ) plus concurrent TTFields. The appended component specifies the adjuvant therapy: 1) CRT-T: CRT followed by adjuvant TMZ alone. 2) CRT-TT: CRT followed by adjuvant TMZ plus continued TTFields. Accordingly, the CRT-T cohort received TTFields concurrently with CRT but discontinued it within one month after completing RT, proceeding with adjuvant TMZ alone. The CRT-TT cohort continued TTFields therapy for more than one month into the adjuvant TMZ phase.

#### Discontinuation of TTFields after concurrent CRT

For patients who discontinued TTFields after completing RT (CRT-T group), the reasons for discontinuation were extracted from clinical notes and treating physician documentation. Reasons were categorized into mutually exclusive categories based on the primary factor documented. These categories included patient-reported adherence burden, financial constraints, scalp toxicity, treating physician recommendation, and other reasons. All discontinuations were voluntary and clinically justified, with no discontinuations due to protocol mandates, consistent with real-world practice.

#### Patient surveillance and follow-up

Contrast-enhanced brain magnetic resonance imaging (MRI) was performed before RT and before adjuvant chemotherapy, and every 2-3 months thereafter (or sooner if suspicious symptom arose). Evaluation of treatment response was according to the Updated Response Assessment Criteria for HGG: Response Assessment in Neuro-Oncology Work Group. Time-to-event endpoints were defined from the date of the definitive surgical procedure (or diagnostic biopsy if no resection). PFS was defined as the time from this date to the first documented disease progression or death from any cause, whichever occurred first. OS was defined as the time from this date to death from any cause. Patients without an event were censored at the date of their last clinical or radiographic assessment. Scalp toxicity was evaluated according to the National Cancer Institute Common Terminology Criteria for Adverse Events (CTCAE, version 5.0).^15^

### Quantification and statistical analysis

Baseline characteristics between the CRT-TT and CRT-T groups were compared using Wilcoxon rank-sum test for non-normally distributed continuous variables and Chi-square or Fisher’s exact test for categorical variables. To account for potential confounding in the non-randomized group comparison, multivariable Cox proportional hazards models were employed to compare PFS and OS between the CRT-TT and CRT-T groups, adjusting for a minimal set of variables selected *a priori* based on clinical prognostic value and univariate screening (*p* < 0.10) to ensure model stability. Specifically: For PFS, the model was adjusted for age (>51 years) and extent of resection (partial resection/biopsy). For OS, the model was adjusted for sex (female), age (>51 years), and extent of resection (partial resection/biopsy). Results are presented as adjusted hazard ratios (aHR) with 95% confidence intervals. No adjustment for multiple testing was applied to subgroup analyses, which are underpowered to detect modest effects.

A sensitivity landmark analysis was implemented to address potential guarantee-time (immortal time) bias arising from the post-baseline definition of treatment groups. Since the decision to continue TTFields into the adjuvant phase occurs after completing concurrent CRT, a landmark was set at 3 months from the date of surgery or biopsy to coincide with the completion of initial therapy. This timepoint ensures that the comparative analysis is restricted to patients who were alive, progression-free, and had completed initial therapy, thus fulfilling the eligibility criteria for the adjuvant treatment decision. Survival outcomes were subsequently compared between the CRT-T and CRT-TT groups starting from this landmark. All statistical analyses were performed using SPSS version 27.0 software (IBM Corporation, Chicago, IL, USA). Visualization of figures was performed using R software (version 4.4.1). For all analyses, p-values < 0.05 were considered statistically significant.
