# Supplementary material for: Tumor treating fields combined with concurrent chemoradiotherapy for glioblastoma: A multi-institutional analysis
Source: iScience. 2026 Jun 23;29(7):116419. doi: 10.1016/j.isci.2026.116419 (PMC13319950; doi:10.1016/j.isci.2026.116419)
Supplement: Document S1. Figure S1 [file mmc1.pdf]

**iScience, Volume *29***

## Supplemental information

# Tumor treating fields combined with concurrent chemoradiotherapy for glioblastoma: A multi-institutional analysis

**Menglan Zhai, Guangyuan Hu, Guoping Shen, Yahua Zhong, Jing Huang, Qianxia Li, Liping Liang, Yong Huang, Jianping Bi, Ying Li, Yanping Li, Guoliang Pi, Hongwei Shi, Hanping He, Vivek Verma, Yang Wang, and Guang Han**

**Fig. S1. Landmark analysis of survival outcomes at 3 months (related to Figure 2).**

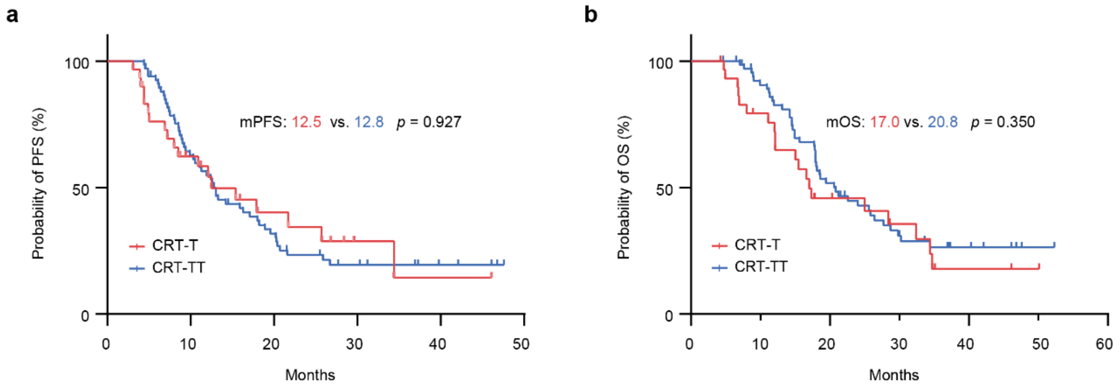


To address potential guarantee-time bias, a landmark analysis was performed at 3 months after surgery or biopsy, excluding 3 patients with early progression (final n = 97). Kaplan-Meier curves show (**a**) PFS and (**b**) OS for the CRT-TT versus CRT-T groups. PFS: $p = 0.927$; OS: $p = 0.350$. PFS, progression-free survival; OS, overall survival.
